# In vitro antioxidant extracts evaluation from the residue of the *Hevea brasiliensis* seed

**DOI:** 10.1038/s41598-021-04017-w

**Published:** 2022-01-10

**Authors:** Giovanna Oleinik, Priscila Paola Dario, Katiane de Morais Gasperin, Dalila Moter Benvegnú, Fernanda Oliveira Lima, Letiére Cabreira Soares, André Lazarin Gallina

**Affiliations:** 1 Chemistry Department, Federal University of Fronteira Sul, Realeza, Paraná 85770-000 Brazil; 2 Chemistry Department, Federal University of Paraná, Curitiba, Paraná 81531-990 Brazil; 3 Graduate Program in Environmental Science, São Paulo State University, Sorocaba, São Paulo 18087-180 Brazil; 4 Chemistry Department, State University of the Midwest, Guarapuava, Paraná 85040-167 Brazil

**Keywords:** Biotechnology, Chemistry

## Abstract

The antioxidants used in the food industry are essential to inhibit the formation of free radicals, preserving the existing properties in the different matrices. However, the insecurity of the synthetic antioxidants regarding human health propels search for natural substrates with potential antioxidant activity as an alternative to synthetic compounds. In this way, the work had as objective obtaining extracts from the seed pomace of the *Hevea brasiliensis* (rubber tree), relating the contents of flavonoids and total phenols in the application as an antioxidant. The methodology consisted of the extraction using four solvents, varying extractive methods, time, and seed concentrations. The antioxidant activity in vitro was evaluated by capturing the DPPH (2,2-diphenyl-1-picryl-hydrazil) radical. The optimized results demonstrate that the aqueous extracts produced in the Soxhlet in the concentrations of 85 g L^−1^ and retention time of 4 h reached 37.73 ± 1.69% in the antioxidant tests of the free radical DPPH capture, 1405.15 mg EAC 100 g^−1^ in the quantification of phenolic compounds and 223.34 mg 100 g^−1^ of total flavonoids. Thus, this work may contribute to the realization of studies and future research for characterization and identification concerning which phenolic compounds and flavonoids attribute the antioxidant characteristic to the extracts produced, enabling the discovery of products with high added value in the production chain. In addition, because the water used as a solvent showed greater antioxidant potential between the extracts, the non-toxic and environmentally friendly character is highlighted, allowing a wide variety of applications in the food industry.

## Introduction

Antioxidants accompany the history of life's emergence on earth. In this way, due to the inappropriate environment with extreme conditions responsible for the free radical formation, the antioxidants were fundamental for the complex molecules constitution^1^. It could capture and stabilize free radicals in the oxidation process, and according to their source, they can be classified as synthetic, petroleum-derived, and natural from biomass^2^.

Synthetic antioxidants are used as preservatives to prolong the products shelf life, highlighting the butylated hydroxyanisole (BHA), the butylated hydroxytoluene (BHT), the tertiary butylhydroquinone (TBHQ), and the propyl gallate (PG), which can exhibit, besides the antioxidant potential by capturing free radicals via hydrogen transfer, the chelating effect of metals. Although widely used by the food industry in Brazil, the application of synthetic antioxidants is argued due to the evidence that these substances' continued consumption can pose health risks^3^.

Currently, in the food sector, the oxidation process of oils and fats poses economic challenges since oxidation leads to the appearance of unpleasant tastes and odors, the reduction of the nutritional properties, and the formation of toxic compounds for humans, which can cause cerebrovascular accidents, and cancer^4^. Despite its use, animal toxicological studies show synthetic antioxidants related to carcinogenic effects, increasingly putting natural antioxidants as an alternative^5^.

Regarding antioxidants from renewable raw materials, industrial by-products and food waste show potential for conversion into bioproducts with antioxidant properties, with the challenges caused by the availability of the material, the degree of toxicity of the extract obtained, and the search for methodologies that allow the greater extraction efficiency^6,7^.

In the Brazilian context, the use of rubber seed bagasse *Hevea brasiliensis* to obtain antioxidant extracts is a promising alternative. The extensive cultivation of the species in Brazil, include the states of Amazonas, São Paulo, Espírito Santo, Mato Grosso, Mato Grosso do Sul, Goiás, Bahia and Paraná^8^. The biorefinery concept could be applied to better use the substrate, which integrates processes for converting the raw material into compounds with added value^9^. With waste generated by the food, beverage, feed, and agriculture industries, the raw material is the most suitable for the biorefinery approach due to the constancy of supply, size, and nutritional content^10^.

From the above, to increase the applications of the same matrix for different purposes, the use of tree seed coproduct from *Hevea brasiliensis* becomes to besides latex exploitation also, an additional value for farmers who cultivate this species. Moreover, aspects such as the circular economy and the maximum use of the matrix during the production chain stages could reduce dependence on fossil sources^6^. Therefore, the concepts of circular economy and bioeconomy come together with common goals of adding value to waste^11^.

The current use of rubber trees consists mainly of extracting the latex used for the production of natural rubber, obtaining the oil from the seeds destined for the paint and varnish industry sector, and, after the latex production cycle, the exploration of the wood^8^. Additionally, there are studies on *Hevea Brasiliensis* latex as a bioactive material in tissue repair in cattle. For this purpose, Nellore cattle were submitted to experimental subcutaneous implants of fragments of natural latex membranes^12^. Seeking to obtain products with greater added value from *Hevea brasiliensis*, Raknam, Pinsuwan, and Amnuaikit^13^ investigated different methods of extracting rubber seed oil as an unconventional oil source for cosmetic products. Fawole et al.^14^ produced a protein isolate from a defatted rubber seed meal. Hassan et al.^15^ performed the characterization of rubber seed bark and rubber seed to make biofuel production. Widyarani et al.^16^ investigated protein and oil production methods from rubber tree seed kernels, focusing on protein recovery. Despite the numerous applications, the literature lacks research exploring the proprieties antioxidant from coproduct of this tree seed.

However, the importance of using plant extracts is a long-standing one, being the first reports of this use as antioxidant related in Ancient Egypt, where one of the factors contributing to the mummification process was this activity attributed to secondary plant metabolites^1^, including vitamins C and E, carotenoids, and phenolic compounds^1,17^. The literature presents numerous studies using different extracts and matrices that correlate the antioxidant activity with the concentration of phenols and flavonoids^18^ and the composition of polyphenols with in vitro antioxidant capacity^19^. In addition, many studies quantify the content of total phenolics^20–24^ and flavonoids in plant matrices^22,25,26^.

From the above, considering the importance of the Hevea brasiliensis species in the Brazilian context, no studies refer to the antioxidant potential of this seed bagasse. Therefore, the challenges concerned synthetic antioxidants and the need to dispose of waste from the production chain with potential for application in rubber tree bioproducts, the present work carried out the study of the optimal extraction conditions for obtaining extracts with in vitro antioxidant potential, considering the presence of total phenols and flavonoids. The varied parameters were different concentrations of rubber seed bagasse, solvents, methods, and extraction times, suggesting apply these antioxidant extracts in the food, pharmaceutical, and bioenergy fields. This study may open paths for research as characterization of extracts with greater potential and identified which phenolic and flavonoid compounds provide this antioxidant characteristic for each extract, valorizing the native species.

## Materials and methods

### Plant materials

The use of plant material in the study complies with relevant institutional, national, and international guidelines and legislation. The samples of rubber seed *Hevea brasiliensis* were collected from the city of Paranaíba, Mato Grosso do Sul, Brazil, by the Kaiser Agropecuária Company, which plants rubber trees. The seeds that fell to the ground were later collected by the company and sent to the Federal University of Fronteira Sul in Realeza. The seeds of *Hevea brasiliensis* would be discarded. However, they were used for research. This research using *Hevea brasiliensis* seed was registered in SisGen (National System for the Management of Genetic Heritage and Associated Traditional Knowledge) under registration number A4C9E3E. The raw material was crushed in a Britannia brand BPM900P multiprocessor to standardize the size of the particles and facilitate oil extraction. Then, the substrate of this extraction called bagasse was used in the experiment.

### Preparation of antioxidant extracts

The extracts were obtained using two extraction methods (infusion and Soxhlet) with different solvents (water, methanol 99.5%, absolute ethanol, and hexane). The solvent was heated to boiling temperature in the first method and then added to the raw material. This mixture was stored in a closed environment, a period described in the experimental planning. For the second method, the sample was inserted into the Soxhlet apparatus^17^.

### Experimental design

The experimental design for the statistical study considered the two extraction methods and four different solvents as descriptive variables. Regarding the quantitative variables, the extraction time and the concentrations of the rubber seed residue were selected, as described in Table 1. The tests were performed in duplicate, and the antioxidant capacity was obtained by the percentage of the capture of the DPPH radical (2,2-diphenyl-1-picryl-hydrazil), determination of flavonoids, and total phenols.

**Table 1:** Levels and variables used in the experimental design.

Variables|levels	− 1	0	+ 1
Extraction time (h)	2	4	6
Extract concentration (g L^−1^)	20	40	60

### Determination of the extracts antioxidant activity

The method for determining the total antioxidant activity consists of the reduction reaction of the DPPH organic free radical, which presents a violet color, forming the yellow-colored compound 2,2-diphenyl-1-picrylhydrazine (DPPH-H). The elimination of DPPH results in a decrease in absorbance (A) at a wavelength of 515 nm^27^.

To carry out this method of determining antioxidant activity, first, the 0.1 mmol L^−1^ DPPH ethanolic solution was prepared and subsequently diluted in ethanol by a factor of 10. The determinations were carried out with the addition of 2.7 mL of DPPH in 0.3 mL of the solvent used in the extract for the control (A_control_).

Regarding the samples, the procedure was repeated. However, instead of the solvent, 0.3 mL of the obtained extract (A_sample_) was added, and, as a blank for the samples, extract, and ethanol was added. The sample blank was used to obtain xr7the final absorbance of the DPPH reduction only. Afterward, the absorbance was analyzed using a Thermo Scientific brand UV/VIS spectrophotometer, model Evolution 201, at a wavelength of 515 nm after 30 min of reaction. All assays were performed in duplicate. The percentage of DPPH free radical capture corresponding to antioxidant activity (AA_DPPH_) was performed based on Eq. (1)^28^.

1 $$ \% AA = \frac{{A_{control} - A_{sample} }}{{A_{control} }}100 $$

where A_control_ is the absorbance of the DPPH control solution and A_sample_ the absorbance of DPPH with the respective sample.

### Determination of the total flavonoids

For total flavonoids quantification, a solution was prepared by adding 0.32 mL of the extract of interest, 0.32 mL of aluminum chloride solution (AlCl_3_), 2% (m/v), and 3.36 mL of ethanol P.A. After 25 min, absorbance was measured in triplicate, through a spectrophotometer at a wavelength of 413 and 427 nm, using 0.32 mL of just sample solvent, 0.32 mL of AlCl_3_ solution and 3.36 mL of ethanol as a blank.

In the analytical curve, the procedure described above was repeated using standard rutin solutions in 60% ethanol, at concentrations from 0.05 to 0.5 mg L^−1^, with a variation of 0.05 mg L^−1^ at each point, to analyze the extracts^29^. The equation of the line was y = 1.32860606x − 0.00746667 with R^2^ = 0.98185.

### Determination of the total phenolics

Phenolic compounds can capture free radicals and metal chelators, working at the beginning of oxidation and the propagation process. According to the methodology described by Sousa et al.^30^ for determining total phenolics in a solution containing 0.1 mL of the extract, 2.5 mL of 0.1 mol L^−1^ Folin Ciocalteau solution and 2.0 mL of saturated sodium carbonate (Na_2_CO_3_) solution were added. To analyze the absorbance of the samples, the tests were stored on a bench for a period of 1 h, and then measurements were performed in triplicate at a wavelength of 720 nm, using water as a blank.

In the construction of the analytical curve, gallic acid was used as a standard at concentrations of 0.1; up to 4.0 mg L^−1^, with variations of 0.5 mg L^−1^, and then, determining the total phenolic content in mg of gallic acid per 100 g^−1^ of the sample^31^, obtaining the equation of the line equal to y = 0.449428x + 0.020706 with R^2^ = 0.99973.

### Physicochemical characterization of extracts: density and pH

According to the values obtained in the statistical analysis of the experimental design, the result with the highest antioxidant capacity was submitted to physical–chemical characterizations. A graduated stem densimeter from Anton Paar, model DMA 35, was used to determine the density of the extracts. The pH of the extracts was measured using a bench-top pH meter brand MS Tecnopon and model mPA 210, previously calibrated.

### Statistical analysis

The t test with a confidence level of 95% performed in the Microsoft Excel^®^ package was used as the first selection criterion for the results related to DPPH radical capture through different extraction methods and extracts. For the initial experimental design analysis, the qualitative variables (solvent and extraction method) and the quantitative variables mentioned in the experimental design were considered.

After verifying the solvent and the method with the greatest antioxidant potential, the response surface methodology was used for the condition that presented the best results, considering a 2^3^ planning using Design Expert^®^ software with the levels and quantitative variables previously described.

In the validation process of the equation represented by the model, the residual dispersion graphs and the analysis of variance table (ANOVA) were considered. The percentage of variation was calculated from the latter, and maximum deviation explained $R^{2}$ the F value of the regression $F_{R}$ and the lack of adjustment $F{}_{la}$, using Eqs. (2), (3), (4) and (5)^32^, respectively. Also, Pearson's correlation test was performed in the Statistica 13 software^®^, where the data were correlated with different methods, time, substrate concentration, % DPPH, flavonoids, and phenols results.

2 $$ R = \frac{{SQ_{R} }}{{SQ_{T} }}100 $$

where $SQ_{R}$ is the ratio between the quadratic sum of the regression and $SQ_{T}$ the total quadratic sum.

3 $$ R^{2} = \frac{{SQ_{T} - SQ_{ep} }}{{SQ_{T} }}100 $$

where $SQ_{ep}$ corresponds to the quadratic sum of the pure error.

4 $$ F_{R} = \frac{{MQ_{R} }}{{MQ_{r} }} $$

where $MQ_{R}$ is the root mean square of the regression and $MQ_{r}$ the root mean square of the residuals.

5 $$ F_{la} = \frac{{MQ_{la} }}{{MQ_{pe} }} $$

where $MQ_{la}$ is the square mean of the regression and the square mean of the residuals $MQ_{pe}$.

## Results and discussions

### Antioxidant activity of extracts using different extraction methods

Table 2 shows the data referring to the mean antioxidant activity of the tests at times of 2, 4, and 6 h, for all extracting solvents and the extraction methods with the t-test application, for comparison of means.

**Table 2:** Results of the t-test, for the percentage of DPPH radical capture, for the different extracting solvents and extraction methods.

Time (h)	Concentration (g L^−1^)	Water^a^	Methanol^b^	Ethanol^a^	Hexane^c^
**Infusion**^**A**^					
2	20	4.16 ± 0.59	8.03 ± 3.39	8.49 ± 0.26	3.51 ± 1.17
2	40	21.68 ± 0.69	–	15.12 ± 0.09	5.55 ± 0.27
2	60	20.78 ± 0.20	9.91 ± 2.07	16.87 ± 0.17	–
4	20	12.54 ± 0.29	–	13.19 ± 0.09	–
4	40	15.24 ± 0.20	1.67 ± 0.74	8.98 ± 0.26	–
4	60	20.85 ± 0.29	2.40 ± 0.44	10.18 ± 0.09	–
6	20	3.05 ± 0.08	2.71 ± 0.15	7.41 ± 0.09	–
6	40	1.45 ± 0.49	6.10 ± 0.22	7.05 ± 0.26	–
6	60	12.67 ± 0.49	8.08 ± 0.66	10.36 ± 0.00	–
**Soxhlet**^**B**^					
2	20	9.35 ± 0.69	8.71 ± 0.81	10.54 ± 0.09	–
2	40	22.65 ± 0.10	19.76 ± 1.40	8.80 ± 0.17	–
2	60	34.00 ± 0.69	13.61 ± 1.25	12.11 ± 0.09	–
4	20	19.18 ± 0.49	12.72 ± 1.92	1.27 ± 1.96	–
4	40	36.36 ± 0.88	13.56 ± 0.74	14.76 ± 1.11	–
4	60	49.45 ± 0.39	10.79 ± 1.11	–	–
6	20	6.58 ± 0.29	10.85 ± 1.03	5.06 ± 2.90	–
6	40	30.13 ± 0.49	12.20 ± 0.15	–	–
6	60	36.43 ± 1.18	19.08 ± 0.30	10.78 ± 0,26	–

– Did not show antioxidant activity.

*Equal letters represent that there was no significant difference between the means, with 95% confidence.

The t test was used to compare the test means for different solvents. Thus, all tests performed with the aqueous solvent and the other solvents were compared, as the aqueous extract was the one that presented the highest average of antioxidant activity. Thus, the t test shows no statistical difference between the solvent water and ethanol for the infusion method. Concerning water and hexane and water and methanol, there was a significant difference.

In the soxhlet method, the water solvent presents a significant difference when compared to all other solvents. Thus, water can be suggested as the best solvent, regardless of the extraction system used, since the average results were higher for this solvent, which is relevant for the study. It is justifiable that aqueous extracts have more significant evolutions since most antioxidant compounds, such as phenolics and flavonoids, are polar, having greater solubility in water. Hence, the extraction with this solvent is more effective. Also, according to Mokrani and Madani^33^, the solubility of phenolic compounds is established according to the polarity of the solvent. That is, each solvent extracts a mixture of phenolic compounds. Flavonoids, for example, are extracted mainly by water, ethanol, or ethyl acetate.

Vizzotto and Pereira^34^ tested solvents with little (hexane) and higher polarity (acetone, methanol, and ethanol). The results obtained by the authors corroborate that less polar solvents presented the lowest effects for the extraction of phenolic compounds, in this case, blackberry. On the contrary, polar solvents were more effective in extracting polyphenols.

Polyphenols and flavonoids can interact whit the DPPH radical by electron ou hydrogen transfer mechanism. The radical specie promotes the homolytic breaking of the H–O bond and generating radicals from polyphenols and flavonoids. The new radicals are stabilized by electronic delocalization along with the aromatic units, interrupting the radical reaction propagation step^27^.

Another observed relationship was temperature since the solvent that came closest to water in terms of antioxidant activity was ethanol (Table 2), which also has a specific polarity and a relatively high boiling point compared to other solvents. It can be suggested high temperatures contribute to the extraction. And several studies have shown results in which elevated temperatures favor the extraction of antioxidant compounds^35–39^.

Regarding the extraction method, according to the t test results, there was a significant difference between Soxhlet and infusion, and the first one presented extracts with a greater antioxidant capacity compared to the infusion results. From the data described in Table 2, it is possible to observe for the two extraction methods, considering the same time, the highest percentage of DPPH capture occurred in the high concentrations of rubber tree seed bagasse, except hexane, which for both methods only showed efficiency for the infusion method at the extraction time of 2 h, with concentrations of 20 and 40 g L^−1^.

The most notable advantages of the soxhlet method are the non-permanent contact of the sample with the solvent, constant renewal, avoiding solvent saturation, and the system temperature remains relatively high, as the heat applied to the evaporation process is continuous. These advantages contribute to higher antioxidant rates extracted in the soxhlet method than the infusion method, which is static and not isothermal^35–39^.

### Optimization of the experimental condition with the highest percentage of DPPH capture

**Figure 1:**
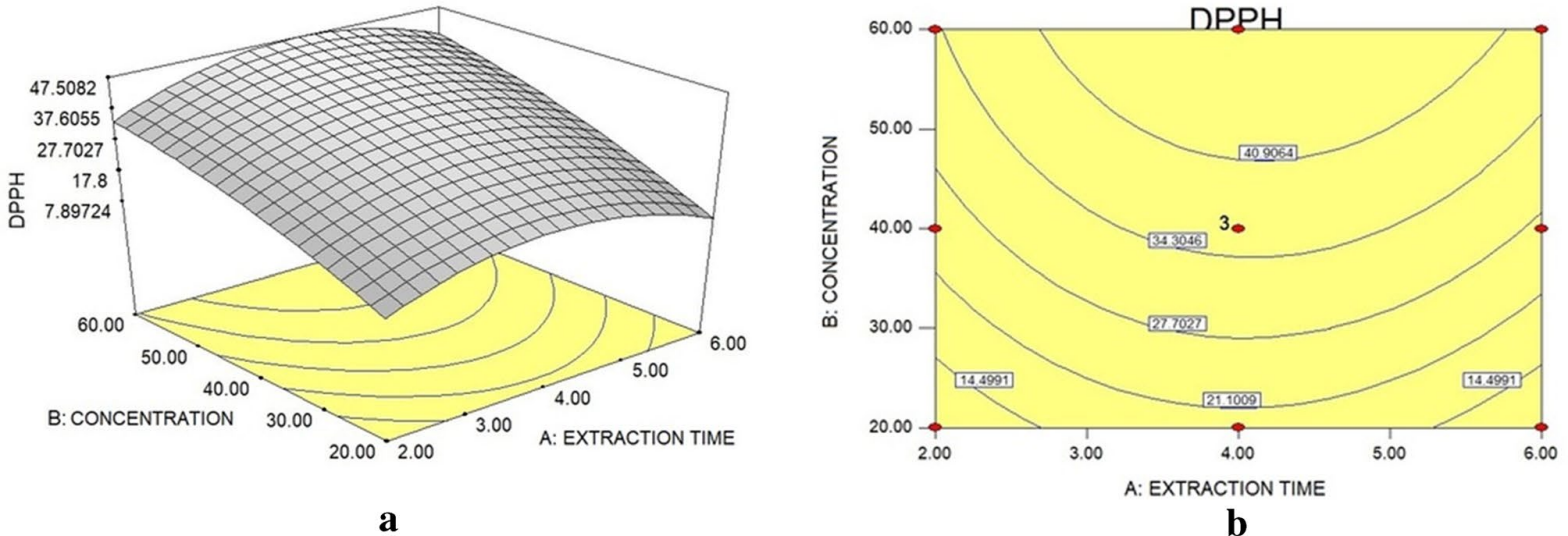
Response surface (**a**) and contour lines (**b**) of the DPPH statistical analysis of the aqueous extract obtained via Soxhlet.

From the conditions established in the first experimental design and the application of the t test to verify the significant difference of samples from solvents and extraction methods, it was found that the best antioxidant activity occurred using water extraction in Soxhlet. Therefore, from the extracts obtained in this condition, the response surface and the contour line graphs presented in Fig. 1a,b were calculated, seeking the pre-optimization of the performed points.

From Fig. 1b, which presents the contour lines, it is possible to observe a tendency to the optimum is using the extraction time from 4 h and extract concentration greater than 40 g L^−1^. In this perspective, according to the response surface to describe the model, Eq. (6) was considered of the quadratic type suggested in the analysis. The ANOVA values are shown in Table 3.

6 $$ {\text{DPPH}}\, = - {46}.{13}\, + \,{21}.{51} \times {\text{Time}}\, + \,{1}.{21} \times {\text{Concentration}}{-}{2}.{78} \times {\text{Time}}^{{2}} - {7}.{91}.{1}0^{{{-}{3}}} \times {\text{Concentration}}^{{2}} \, + \,0.0{32} \times {\text{Time}} \times {\text{Concentration}} $$

**Table 3:** ANOVA for the results statistical treatment related to the planning for obtaining extracts by Soxhlet in aqueous solvent.

Factor	Quadratic sum (SQ)	Degrees of freedom (DF)	Square mean (MQ)	F	*P*
Regression (R)	1628.02	5	325.60	48.52	0.0003
Time (linear)	8.50	1	8.50	1.27	0.3116
Time (quadratic)	312.46	1	312.46	46.56	0.0010
Concentration (linear)	1197.94	1	1197.94	178.53	< 0.0001
Concentration (quadratic)	25.39	1	25.39	3.78	0.1093
Interaction time × concentration	6.73	1	6.73	1	0.3624
Residue (r)	33.55	5	6.71		
Lack of adjustment (la)	30.06	3	10.02	5.75	0.1518
Pure error (ep)	3.49	2	1.74		
Total quadratic sum (SQ_T_)	1661.57	10			

In the validation process of the model equation for the response surface, the percentage of maximum variation explained by the quadratic equation corresponded to R = 99.79%, while the obtained variance presented R^2^ = 97.98%, allowing a predictive capacity with relation to the trend of the experimental points for the set of variables considered in the experiment.

Another aspect considered is the statistical significance presented by the values F_R_ = 48.52 and F_la_ = 5.76 for the reference for the percentage of the F 5% distribution, corresponding to 5.05 and 19.16 respectively, the first being much higher and the second smallest about the value of F_R_ and F_la_ obtained in the model^32^.

**Figure 2:**
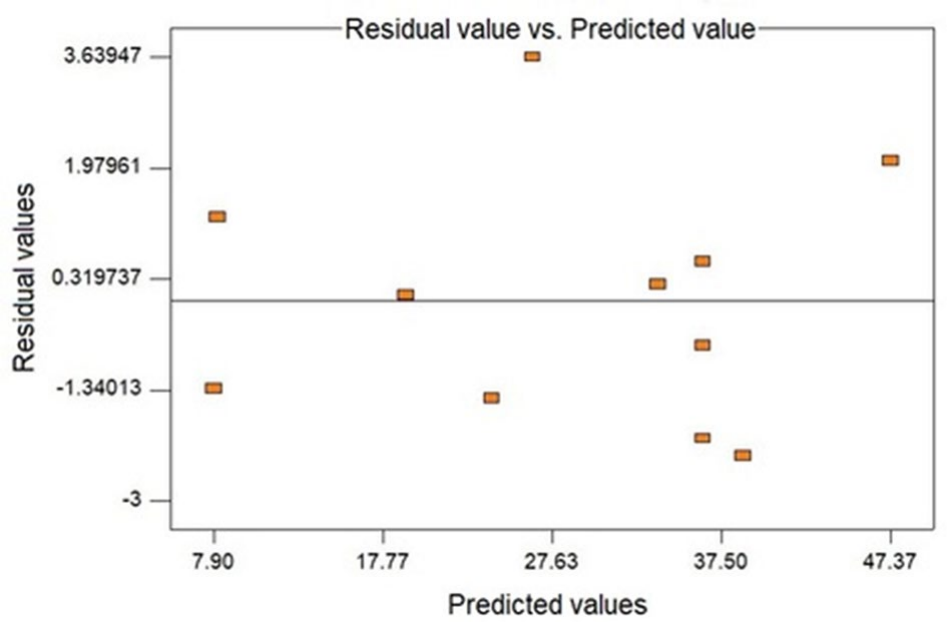
Plot of residuals versus predicted for the regression of the DPPH statistical analysis of the aqueous extract obtained via Soxhlet.

The graph presented in Fig. 2 describes the random dispersion of the residuals, concluding that the quadratic equation used to represent the response surface according to the conditions used does not follow a trend. Therefore, a second analysis was performed looking for the path of the improved condition to obtain a greater antioxidant capacity of the extract from the aqueous solution using the Soxhlet system^24,40–42^.

**Figure 3:**
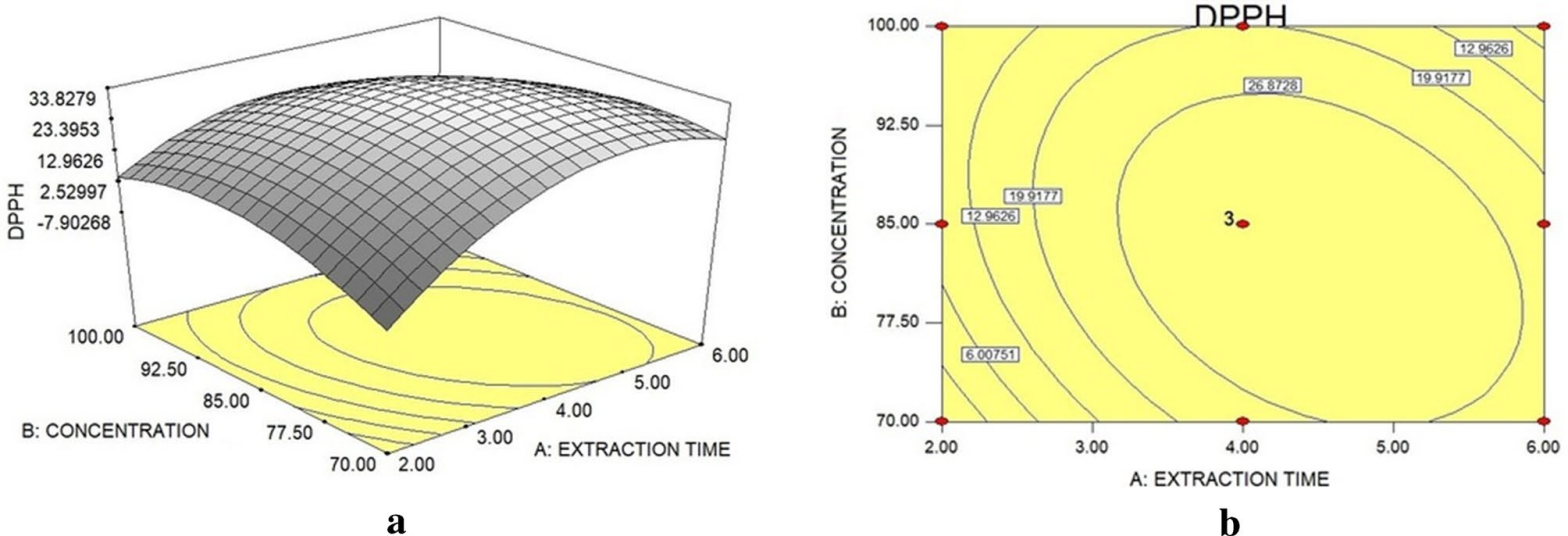
Response surface (**a**) and contour lines (**b**) of the DPPH statistical analysis of the aqueous extract obtained via Soxhlet for extraction optimization.

From the results obtained in the first response surface, the path to the optimum happened through the second experimental design with the same time values but increased concentrations to 70, 85, and 100 g L^−1^. According to the methodology described for the generated response surface, data analysis is presented in Fig. 3a and the contour lines graph in Fig. 3b.

The result for the best antioxidant activity of the rubber tree seed bagasse present in the extract corresponded to 85 g L^−1^, with an extraction time of 4 h. Under these conditions, the percentage of antioxidant activity via DPPH capture was 37.73 ± 1 0.69%. The fact that the highest antioxidant capacity value is obtained for an intermediate concentration can be explained by the extraction method, which consists of a purely physical process, with no chemical reaction in getting the extract, that is, the extractive release process by the penetration of the solvent into the plant matrix is one of the determining factors in the extraction^43^. Therefore, one of the hypotheses is that the increase of the vegetal matrix above the concentration of 85 g L^−1^ may have affected the penetration of the solvent and, consequently, decreasing the concentration of antioxidant compounds.

Also, regarding the second planning, the ANOVA results are shown in Table 4, and Eq. (7) describes the quadratic model for the behavior of data according to the variables in the analysis.

7 $$ {\text{DPPH}}\, = - {471}.0{6}\, + \,{59}.{68} \times {\text{Time}}\, + \,{9}.00 \times {\text{Concentration}}{-}{4}.{21} \times {\text{Time}}^{{2}} - 0.0{5} \times {\text{Concentration}}^{{2}} {-}0.{27} \times {\text{Time}} \times {\text{Concentration}} $$

**Table 4:** ANOVA for the statistical treatment of the results related to the planning to obtain the optimization of the extraction.

Factor	Quadratic sum (SQ)	Degrees of freedom (DF)	Square Mean (MQ)	F	*P*
Regression (R)	1923.01	5	284.60	5.10	0.0491
Time (linear)	305.31	1	305.31	4.05	0.1005
Time (quadratic)	716.76	1	716.76	9.50	0.0274
Concentration (linear)	21.55	1	21.55	0.29	0.6160
Concentration (quadratic)	289.26	1	289.26	3.83	0.1076
Interaction time × concentration	251.70	1	251.70	3.33	0.1274
Residue (r)	377.37	5	75.47		
Lack of adjustment (la)	362.78	3	120.93	16.58	0.0574
Pure error (ep)	14.59	2	7.29		
Total quadratic sum (SQ_T_)	2300.38	10			

In the model validation for the response surface, R^2^ showed 83.60% confidence, while the maximum variation reached 99.37%. In this context, the other parameters were evaluated, seeking greater reliability in predicting the results. Regarding the F_R_ and F_la_ values, the values found corresponded to 5.10 and 16.59. Thus, considering the percentage of the F distribution equal to 5%, these values show statistical significance.

**Figure 4:**
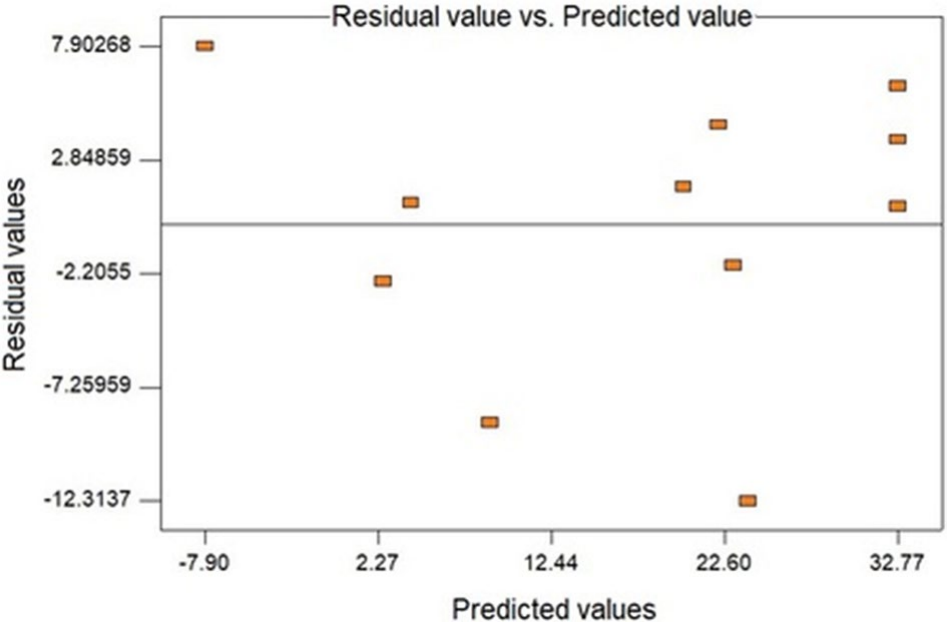
Graph of residues versus predicted for the regression of the DPPH statistical analysis, of the aqueous extract obtained via Soxhlet for the second planning developed.

The last parameter considered the residue scatter plot (Fig. 4), where random behavior was observed. Therefore, from the evaluation carried out, it is possible to conclude that the second model presents an adjustment to the variables selected for the analysis but with lower reliability when compared to the first planning.

According to Sousa^31^, percentages lower than 50% of DPPH free radical capture are classified as weak antioxidant activity. Therefore, considering that the highest percentage of DPPH capture achieved in the second planning was 37.73%, the extracts produced with the seed of *Hevea brasiliensis* have weak antioxidant activity. However, it is essential to highlight that the substrate used to carry out the extracts is the coproduct of this tree seed.

About the literature, the study by Zain et al.^44^ found a value of 34.84% of DPPH free radical capture for methanol extract of seeds of the RRIM 2025 rubber tree clone *Hevea brasiliensis*. Machado et al.^22^ found for *Garcinia cochinchinensis Choisy* fruit extracts 90.6 ± 2.52% of DPPH capture. Bryan-Thomas^45^ found 46.24% of antioxidant activity for *M. zapota* extract and 9.39% for *A. muricata* extract. Bispo et al.^46^ evaluated the antioxidant capacity of coffee wood extracts, and the Catuaí variety showed the best results, with 34.5% of DPPH sequestration. Pereira et al.^47^ evaluated the antioxidant profile of a mixed juice containing kale (*Brassica oleracea *L.), yam (*Dioscorea *spp.), and orange (*Citrus sinensis*) and presented a sequestration percentage of 94.81%. Da Silva Acácio et al.^48^ found a percentage of 84.89% of DPPH free radical capture for the extract of *Melochia tomentosa L*. at a concentration of 75 μg mL^−1^.

The antioxidant activity of rubber tree seed bagasse is weak but has higher or similar values than some other matrices, being those seeds and fruits. Considering there are no reports in the literature of using this material as an antioxidant and the low cost of acquiring the raw material because it is derived from an oil extraction process, the indices found are satisfactory and promising, adding value in the production chain.

### Determination of the total phenolics and flavonoids concentration

#### Total phenolics and flavonoids from the first planning

**Figure 5:**
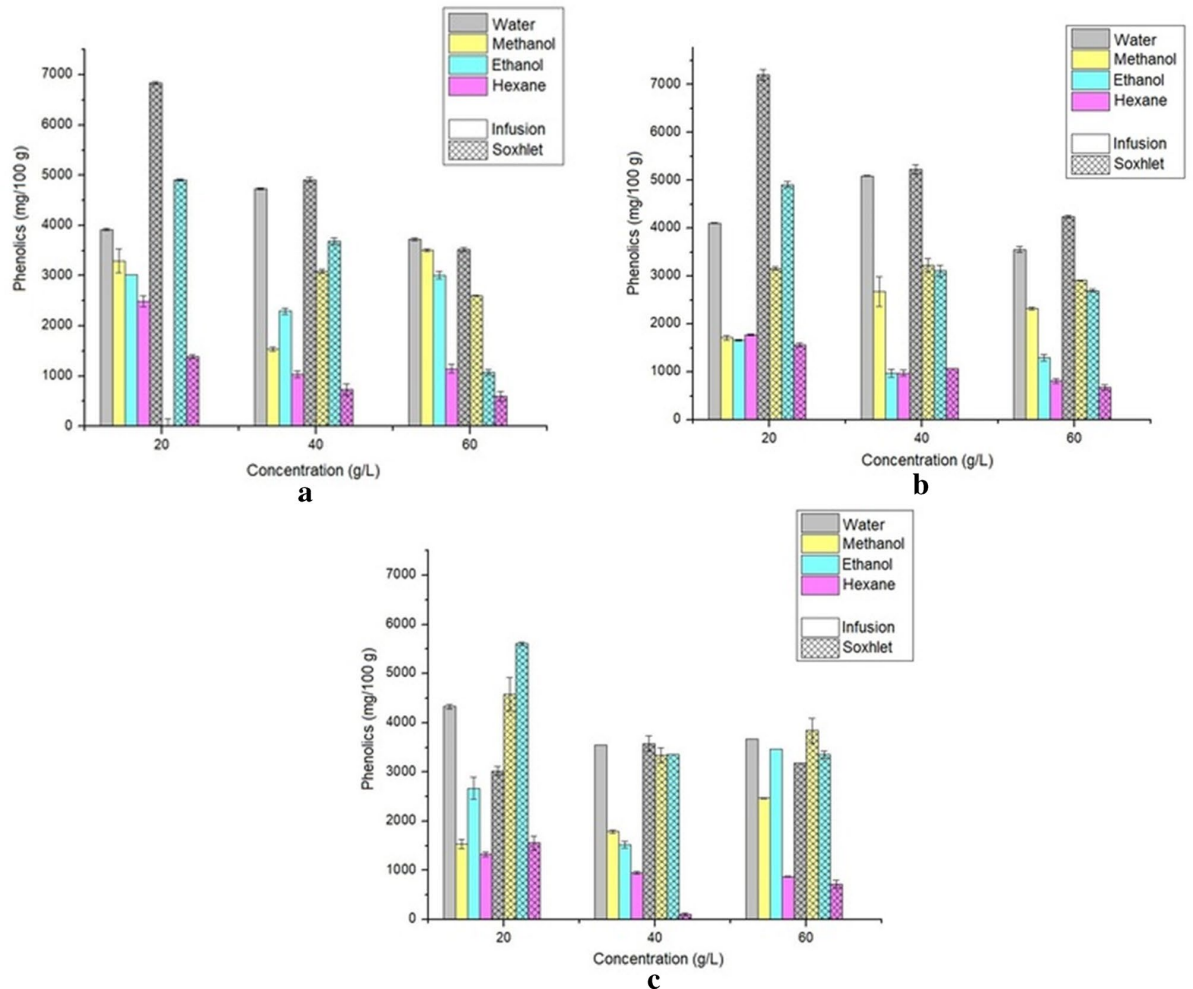
Concentration of total phenols using 2 h (**a**), 4 h (**b**), and 6 h (**c**) as extraction time.

The evaluation of the total phenolic concentration is shown in Fig. 5, where the different methods, solvents, and concentrations were analyzed according to each extraction time.

Compared with other works performed in the literature on the species, the content of total phenolic compounds is satisfactory, although there are no studies that analyze the antioxidant potential of *Hevea brasiliensis* seed bagasse. The extracts in this study reached 7201.32 mg of gallic acid equivalents 100 g^−1^ (mg GAE 100 g^−1^) in the aqueous extract in the Soxhlet, in the concentration of 20 g L^−1^ and 4 h of extraction, while Agbai et al.^49^ found for the sample of raw rubber seed meal *Hevea brasiliensis* (RRSM) a content of phenolic compounds of 2.77 ± 0.06 mg GAE g^−1^. Ismun et al.^50^ determined the polyphenol content in *Hevea brasiliensis* latex serum C and in the effluent for rubber processing, finding a 0.0393 g GAE mL^−1^ in latex serum C and a content of 0.0099 g GAE mL^−1^ in the effluent. Zain et al.^44^ found content of 0.010 mg GAE mL^−1^ of phenolic compounds for the methanol extract of seeds of the rubber tree clone RRIM 2025 *Hevea brasiliensis*.

Concerning other studies with different matrices, Sarkis^23^ obtained 690 mg GAE 100 g^−1^ of total phenolics for pecan extract, using a water/ethanol solution as solvent (20:80, v/v). Wang et al.^24^ found 92.96 ± 1.47 mg of GAE g^−1^ of phenolic compounds for nutshell ethanol extracts. Da Silva et al.^21^ evaluated the total phenols for jilo extracted with different solvents, and the optimized result was 830.6 ± 16.2 mg GAE 100 g^−1^ with extraction with 20% methanol/water (v/v). Alasalvar and Bolling^20^ obtained a total of 112–310 mg 100 g^−1^ for Brazil nuts, 137–274 mg 100 g^−1^ for cashew, 47–418 mg 100 g^−1^ for almonds, 46–156 mg 100 g^−1^ for Macadamia and 1285–2016 mg 100 g^−1^ for Pecan nut. Machado et al.^22^ found extracts (with 80% acetone) of the pulp of *Garcinia cochinchinensis Choisy* a total phenolic content of 469.6 ± 114.9 mg gallic acid 100 g^−1^, and in leaves 3739.7 ± 310.5 mg gallic acid 100 g^−1^.

According to Assis et al.^51^, phenolic compounds, particularly phenolic acids, are found mainly in higher polarity extracts. They investigated the extraction of phenolic compounds from the microalgae *Spirulina platensis* and *Chlorella pyrenoidosa* with methanol and ethanol solvents, and the methanol extracts from the two microalgae showed higher content of phenolic compounds (MES (methanolic extracts of Spirulina) = 2.62 mg GAE g^−1^; MEC (methanolic extracts of Chlorella) = 0.69 mg GAE g^−1^) compared to ethanol extracts (EES (ethanolic extracts of Spirulina) = 1.37 mg GAE g^−1^; EEC (ethanolic extracts of Chlorella.) = 0.41 mg GAE g^−1^). Manivannan et al.^52^ studied methanol, diethyl ether, and hexane solvents to extract phenolic compounds from the microalgae *Chlorella marina* and obtained the best results for methanol extracts (0.64 mg GAE g^−1^). Hajimahmoodi et al.^53^ found for *Chlorella vulgaris*, 3.69–19.14 (mg GAE g^−1^) for the aqueous fraction, 0.02–3.59 (mg GAE g^−1^) for the aceto-ethyl fraction and 0.02–0.49 (mg GAE g^−1^) for the hexane fraction. These results corroborate the results presented, which indicated the polar solvents extracted more phenolic compounds.

**Figure 6:**
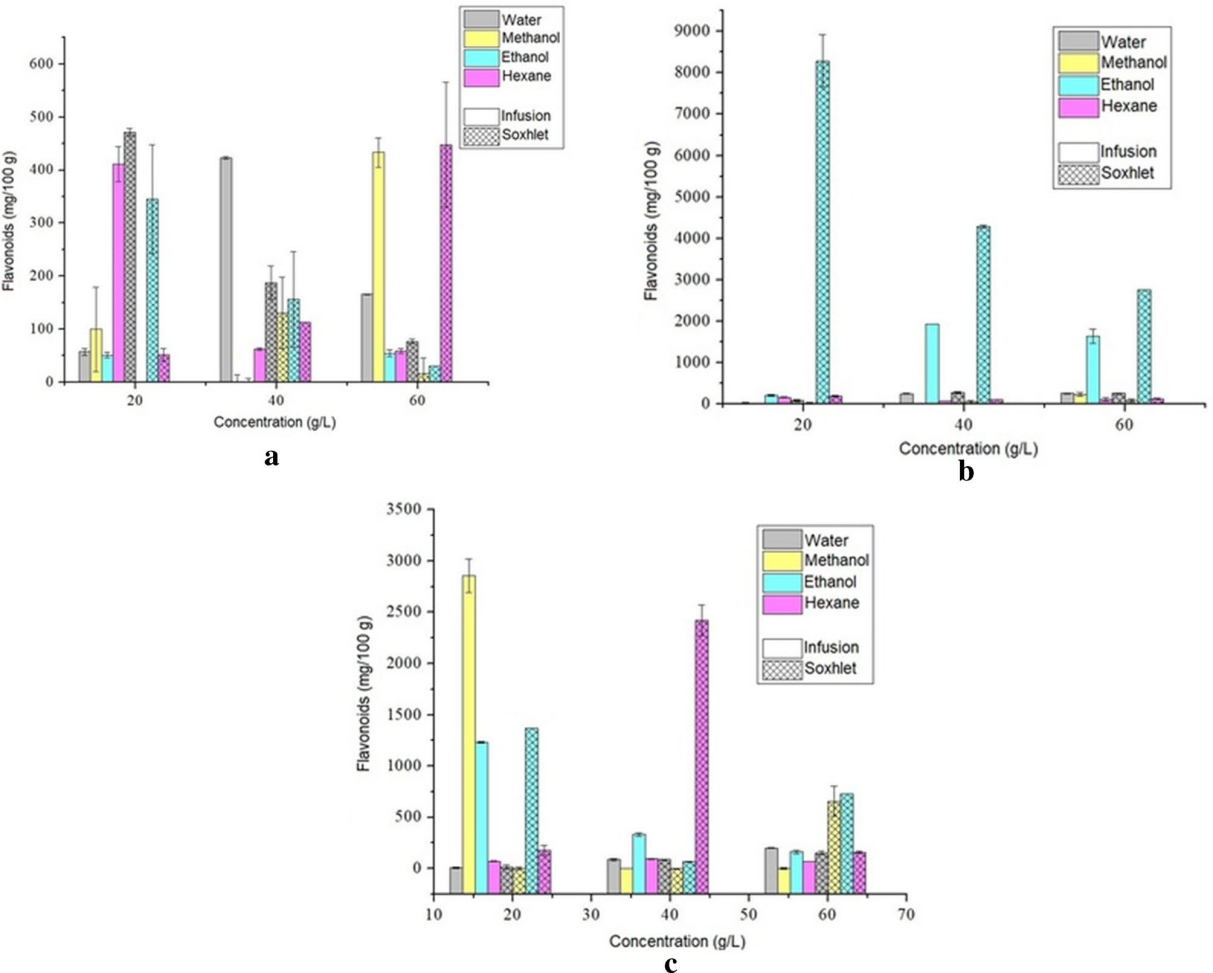
Total flavonoids concentration using 2 h (**a**), 4 h (**b**), and 6 h (**c**) as extraction time.

Figure 6 shows the different methods, solvents, and concentrations analyzed according to each extraction time to flavonoids.

According to the results obtained, the maximum concentration of total flavonoids was extracted using the soxhlet method in 4 h and with the ethanol solvent, reaching 8290.52 mg of rutin 100 g^−1^. For the aqueous extract, the best extraction method was Soxhlet at a concentration of 20 g L^−1^ and 2 h, with a value of 471.23 mg of rutin 100 g^−1^. The extract from the first planning, which showed more significant activity in DPPH capture (4 h of extraction, Soxhlet, 60 g L^−1^, water as solvent), has a significantly higher concentration of phenolic compounds (4241.67 mg GAE 100 g^−1^) in the composition of the extract compared to the concentration of flavonoids (254.92 mg of rutin 100 g^−1^). Thus, the capture of DPPH was almost exclusively due to the activity of phenolic compounds. The phenolic compounds obtained during plant extracts have different structures, such as phenolic acids, coumarins derivatives, tannins, and flavonoids^54^.

It is possible to verify, in the present work, flavonoid quantification results superior to those of Agbai et al.^49^, which were obtained for the sample of raw rubber seed meal (RRSM) *Hevea brasiliensis* a flavonoid content of 60.00 ± 3.53 mg quercertin 100 g^−1^. Zain et al.^44^ found 0.200 mg of catechin mL^−1^ for methanol extract with leaves of the rubber tree clone RRIM 3001 *Hevea brasiliensis*. Nevertheless, it did not find flavonoids for methanol extracts with rubber tree clone seeds. Gullón et al.^25^ presented for purple corn *Zea mays L*. 18.6 mg of rutin g^−1^ of flavonoids. Rico et al.^26^ found a flavonoid content of peanut shell self-hydrolysis liqueurs of 10.30 mg of rutin.g^−1^ of raw material. Machado et al.^22^ presented for *Garcinia cochinchinensis Choisy* leaf extract a flavonoid content of 665.1 ± 122.9 mg of rutin 100 g^−1^. The presence of flavonoids is an indication of the improvement in antioxidant activity^55^. However, as the rubber tree seed pomace is a complex matrix, synergistic activity could happen between the antioxidant compounds.

Using the t test to verify the statistical difference in the concentration of phenols and flavonoids, the extraction methods using infusion and Soxhlet with aqueous solvent were considered, at concentrations 40 g L^−1^ and 60 g L^−1^, these tests being related to the higher DPPH values found in Table 2. The results showed that the extraction methods do not differ statistically for the contents of phenols and flavonoids for these conditions. However, for the latter, in the soxhlet system, the difference was significant between the concentrations (p = 0.0169). For the concentration of 60 g L^−1^, the average was higher, corroborating the choice for the development of the second planning, the increase in concentrations.

#### Total phenolics and flavonoids from second planning

Afterward, a second analysis was performed from the condition that showed the best antioxidant activity, corresponding to 85 g L^−1^ and the concentration of 100 g L^−1^. At this concentration, the phenolic compounds content was 1405.17 mg GAE 100 g^−1^, and the flavonoid content 223.34 mg 100 g^−1^. Statistically, differences were found between the content of phenols (p = 0.024) and flavonoids (p = 0.0013). For both compounds, the content was higher in the experiment containing 85 g L^−1^ and 4 h of extraction, which is correlated with the antioxidant capacity data using the DPPH. As in the initial planning, the phenols content was higher than flavonoids, following the same behavior already demonstrated, suggesting that the antioxidant capacity is linked to the phenols content or synergism of the compounds in the sample.

### Pearson correlation

Person correlation tests were performed to verify the existence of a linear correlation between the variables and results obtained for each solvent. In this way, the Pearson correlation for aqueous solvent is shown in Table 5.

**Table 5:** Pearson correlation for aqueous solvent.

	Method	Concentration	Time	% DPPH capture	Flavonoids	Phenolics
Method		0.00	− 0.00	0.5647*	0.0727	0.2489
		p = 1.00	p = 1.00	p = 0.015*	p = 0.774	p = 0.319
Concentration	0.00		0.00	0.6265*	0.2410	− 0.4536
	p = 1.00		p = 1.00	p = 0.005*	p = 0.335	p = 0.059
Time	− 0.00	0.00		− 0.1172	− 0.4310	− 0.3821
	p = 0.00	p = 1.00		p = 0.643	p = 0.074	p = 0.118
% DPPH capture	0.5647*	0.6265*	− 0.1172		0.2626	− 0.0458
	p = 0.015*	p = 0.005*	p = 0.643		p = 0.293	p = 0.857
Flavonoids	0.0727	0.2410	− 0.4310	0.2626		0.4482
	p = 0.774	p = 0.335	p = 0.074	p = 0.293		p = 0.062
Phenolics	0.2489	− 0.4536	− 0.3821	− 0.0458	0.4482	
	p = 0.319	p = 0.059	p = 0.118	p = 0.857	p = 0.062	

*Results statistically significant.

As shown in Table 5, most variables do not demonstrate a linear correlation with the results of flavonoids and phenolics for the aqueous extract, except for the % DPPH capture, which showed a median and positive correlation statistically significant with the variables method and concentration. These results indicate that the Soxhlet method contributes to the greater extraction of antioxidant compounds responsible for capturing free radicals or their non-degradation. By this method, the solvent does not come into contact with the sample at the boiling temperature. This ability to neutralize free radicals cannot be related to phenolic compounds or phenols by this analysis. Pearson's correlation analysis was also performed for the methanol extract, and the results are shown in Table 6.

**Table 6:** Pearson correlation for methanol extract.

	Method	Concentration	Time	% DPPH capture	Flavonoids	Phenolics
Method		0.00	− 0.00	0.7927*	− 0.2274	0.3204
		p = 1.00	p = 1.00	p = 0.000*	p = 0.364	p = 0.195
Concentration	0.00		0.00	0.2459	− 0.1597	0.2239
	p = 1.00		p = 1.00	p = 0.325	p = 0.527	p = 0.372
Time	− 0.00	0.00		− 0.0119	0.2944	0.2341
	p = 0.00	p = 1.00		p = 0.963	p = 0.236	p = 0.350
% DPPH capture	0.7927*	0.2459	− 0.0119		− 0.1505	0.5475*
	p = 0.000*	p = 0.325	p = 0.963		p = 0.551	p = 0.019*
Flavonoids	− 0.2274	− 0.1597	0.2944	− 0.1505		− 0.1575
	p = 0.364	p = 0.527	p = 0.236	p = 0.551		p = 0.532
Phenolics	0.3204	0.2239	0.2341	0.5475	− 0.1575	
	p = 0.195	p = 0.372	p = 0.350	p = 0.019	p = 0.532	

*Results statistically significant.

In Table 6, as well as in the aqueous extract, the methanol solvent extract presented a linear correlation with the result of % DPPH capture, which is a statistically significant, high, and positive correlation with the employed method. For this extract, it was also observed that the Soxhlet method contributes to the increase in DPPH capture. This ability to neutralize free radicals can be related to phenolic compounds by this analysis, as there was a statistically significant and positive correlation between % DPPH capture and the amount of phenols. Table 7 shows the results of the Pearson correlation for the extract obtained in the ethanol solvent.

**Table 7:** Pearson correlation for the ethanol extract.

	Method	Concentration	Time	% DPPH capture	Flavonoids	Phenolics
Method		0.00	− 0.00	− 0.3984	0.3398	0.5478*
		p = 1.00	p = 1.00	p = 0.101	p = 0.168	p = 0.019*
Concentration	0.00		0.00	0.2038	− 0.2046	− 0.4127
	p = 1.00		p = 1.00	p = 0.417	p = 0.415	p = 0.089
Time	− 0.00	0.00		− 0.4445	0.1088	0.1036
	p = 0.00	p = 1.00		p = 0.065	p = 0.667	p = 0.683
% DPPH capture	− 0.3984	0.2038	− 0.4445		− 0.3778	− 0.3157
	p = 0.101	p = 0.417	p = 0.065		p = 0.122	p = 0.202
Flavonoids	0.3398	− 0.2046	0.1088	− 0.3778		0.2954
	p = 0.168	p = 0.415	p = 0.667	p = 0.122		p = 0.234
Phenolics	0.5478*	− 0.4127	0.1036	− 0.3157	0.2954	
	p = 0.019*	p = 0.089	p = 0.683	p = 0.202	p = 0.234	

*Results statistically significant.

There is a linear correlation between the extraction method and the amount of phenols for the ethanol solvent, and this correlation is statistically significant, positive, and intermediate level. This result indicates that the Soxhlet method amplifies the amount of phenols extracted by this solvent, enhancing the antioxidant capacity. Also, Table 8 shows the Pearson correlation for the hexane extract.

**Table 8:** Pearson correlation for the hexane extract.

	Method	Concentration	Time	% DPPH capture	Flavonoids	Phenolics
Method		0.00	− 0.00	− 0.3439	0.2805	− 0.3191
		p = 1.00	p = 1.00	p = 0.162	p = 0.168	p = 0.197
Concentration	0.00		0.00	− 0.1632	− 0.0107	− 0.6927*
	p = 1.00		p = 1.00	p = 0.518	p = 0.967	p = 0.001*
Time	− 0.00	0.00		− 0.4212	0.2360	− 0.2440
	p = 0.00	p = 1.00		p = 0.082	p = 0.346	p = 0.329
% DPPH capture	− 0.3439	− 0.1632	− 0.4212		− 0.0479	0.3321
	p = 0.162	p = 0.518	p = 0.082		p = 0.850	p = 0.178
Flavonoids	0.2805	− 0.0107	0.2360	− 0.0479		− 0.4004
	p = 0.168	p = 0.967	p = 0.346	p = 0.850		p = 0.100
Phenolics	− 0.3191	− 0.6927*	− 0.2440	0.3321	− 0.4004	
	p = 0.197	p = 0.001*	p = 0.329	p = 0.178	p = 0.100	

*Results statistically significant.

According to the Pearson correlation statistical test for the hexane solvent, the only statistically significant linear correlation was phenols with concentration. There is a minimization of the amount of phenols when increasing the study concentration, indicating that hexane is not the ideal solvent for extracting phenolic compounds for this biomass.

### Physicochemical characterization of the extract: density and hydrogenic potential

The physicochemical analyzes were carried out considering the extract with the most significant antioxidant potential was the aqueous extract at the concentration of 85 g L^−1^, in a time of 4 h. For the sample, the pH and density (ρ) values reached 6.21 and 0.999 g mL^−1^, respectively, at a temperature of 25 °C, values close to those found in the literature for similar plant species. For example, Cardoso et al.^56^ found pH indices for aqueous extracts of Brazil nuts of 6.34 without preservatives and 5.87 with added preservatives.

## Conclusions

Brazil's rubber tree has been explored for latex production, but few studies about other commercial applications are described in the literature, mainly for its residues. This work showed that the aqueous extracts, made from rubber tree seed bagasse residue, are rich in phenolic compounds and flavonoids, superior to several antioxidant extracts produced from other seeds showed in other works during the paper. The results of statistical correlation showed that DPPH radical capture, reaching 37.73%, is closely linked to the presence of phenols, attributed to ease of extraction by aqueous solvent. It was evidenced that the method Soxhlet is one of the variables that most correlates with the antioxidant activity, mainly to DPPH, thus suggesting that it is suitable for extracts that intend to be used as antioxidants. The antioxidant activity allows the application of the extract in different industry sectors, contributing to future research on the characterization and identification of phenolic compounds and flavonoids responsible for the activity of the antioxidant extract. Besides that, the valorization of this industrial coproduct makes the productive chain economically and environmentally attractive, favoring the development of integrated processes at the laboratory, pilot, and industrial scale.

## Data Availability

The datasets generated during and/or analyzed during the current study are available from the corresponding author on reasonable request.
